# Effect of amaranth leaf meal on performance, meat, and bone characteristics of Ross 308 broiler chickens

**DOI:** 10.1371/journal.pone.0271903

**Published:** 2022-08-09

**Authors:** Tlou Grace Manyelo, Nthabiseng Amenda Sebola, Monnye Mabelebele

**Affiliations:** 1 Department of Agriculture and Animal Health, College of Agriculture and Environmental Sciences, University of South Africa, Pretoria, South Africa; 2 Department of Agricultural Economics and Animal Production, University of Limpopo, Sovenga, South Africa; Bangabandhu Sheikh Mujibur Rahman Agricultural University, BANGLADESH

## Abstract

This study aimed to determine the effect of amaranth leaf meal (ALM) inclusion levels on the productivity of Ross 308 broiler chickens. A total of 200, day-old, Ross 308 broiler chicks were randomly allocated to five dietary treatments in a complete randomized design, with each group having four replicates with ten chicks each. Amaranth leaf meal inclusion levels used in this study were 0, 5, 10, 15, and 20%. Bodyweight and feed intake were measured weekly to calculate the feed conversion ratio. Meat and bone traits of broiler chickens fed amaranth leaf meal at varying levels were also measured whereas the general linear model of statistical analysis software was used to analyze collected data. Amaranth leaf meal inclusion level had no effect (p > 0.05) on initial weight, final weight, average daily gain, and average daily feed intake of Ross 308 broiler chickens aged one to 42 days. Ross 308 broiler chickens which were given diets with 0 and 10% ALM inclusion levels, had higher (p < 0.05) cooking loss than those on diets with 5, 15, and 20% of ALM inclusion levels. Chickens fed with a diet having a 15% ALM inclusion level, had a greater (p < 0.05) tibia diameter than those on 0, 5, 10, and 20% ALM inclusion levels. Ross 308 broiler chickens which were fed with 5 and 15% ALM inclusion levels, had larger (p < 0.05) femur diameters than those on 0, 10, and 20% ALM inclusion levels. Ross 308 broiler chickens fed with diets having a 15% ALM inclusion level, had longer (p < 0.05) tibia lengths than those fed with diets having 0, 5, and 10% ALM inclusion levels. It can be concluded that amaranth leaf meal can be included in the diets of Ross 308 broiler chickens aged one to 42 days at 5, 10, and 15% levels without adverse effects on productivity, meat quality, and the bone characteristics of the chickens. This augurs well for poultry farmers in many parts of Africa where the production of vegetable amaranth is in abundance.

## 1. Introduction

The burden of high feed costs in the poultry sector calls for deeper exploration of underutilized alternatives that are rich in nutrients and have the potential to reduce livestock feed prices. Indigenous vegetables such as amaranth crops can play a significant role in the reduction of poultry expenses. The probable use of amaranth inclusion in poultry diets as a protein source has remained a subject of interest in scientific research [1]. Amaranth is considered one of the most produced and consumed indigenous vegetables on the African continent with high nutritional capacities but is yet to be fully exploited. According to [1, 2] underutilized vegetables such as amaranth are promising protein sources because it is drought tolerant and thus can grow in a wide range of weather conditions [1, 3]. According to [4] amaranth vegetable is one of the species grown primarily for grains, although, its leaves are highly recommended to be consumed when they are still tender. Amaranth species have appreciable amounts of minerals and amino acids with high content of sulfur-containing amino acids such as methionine and cysteine [5, 6], which make this vegetable to be suitable for use in the formulation of complementary poultry feeds. Formulation of diets that meet nutrient levels for optimal productivity of improved broiler chickens has lagged and that has resulted in skeletal problems, particularly in leg bones. According to [7], the skeletal integrity of broiler chickens has become a welfare and economic concern. About 1.1% of chickens have been associated with weak bones which are undesirable traits in growing broiler chickens. However, several studies have been conducted to determine the physical (length, diameter) and mineral contents (Ca and P) of femur and tibia bones of broiler chickens when fed different leaf meals [7, 8]. Moreover, several studies have been conducted on the determination of amaranth vegetables on the meat quality of chickens. According to [9, 10] amaranth meal did not affect the meat characteristics of broiler chickens. However, other authors observed an increase in the breast muscle percentage in carcasses of chickens fed with an 8% amaranth feed mixture [11]. However, the authors stated that an increase in breast muscle might be due to 3% flax oils, which were part of their formulated diet. [12] reported an increase in abdominal fats when chickens were fed diets having an amaranth leaf meal inclusion of up to 10%. According to [13] amaranth meal did not affect the digestibility of nutrients and bird growth rates of Hybro N and Isa Brown laying hens. However, the difference in the performance, meat quality and physical characteristics of broiler chickens fed vegetable amaranth remains inconclusive. Moreover, to date, no study has established a suitable and optimal level of inclusion of amaranth leaf meal in chicken diets. Therefore, the objective of this study is to then establish and determine an amaranth leaf meal inclusion level and that improves the performance, meat quality, and bone characteristics of Ross 308 broiler chickens.

## 2. Materials and methods

### 2.1 Study site

The study was conducted at the University of Limpopo Animal unit (latitude of 27.55° S and longitude of 24.77° E). The ambient temperature at the study site ranges from 20 to 36°C in the summer months (November—January) and from 5 to 25°C in the winter months (May–July). Mean annual rainfall ranges between 446.8 and 468.44 mm. This study was conducted during the winter.

The experimental procedures were conducted in accordance with the University of South Africa’s (UNISA) Ethics code for the use of live animals in research, Ethics Reference Number: 2019/CAES_AREC/154, and the University of Limpopo (UL) Ethics Committee, Reference Number: AREC/12/2020: IR.

### 2.2 Experimental diets, design, and procedures

A total of 200, day-old, male Ross 308 broiler chicks with an initial live weight of 42±8 g/bird, were received from a local hatchery and randomly allocated to five dietary treatment levels in a complete randomized design, with a treatment group having four replicates and ten chicks each. Amaranth leaf meal inclusion levels were at 0, 5, 10, 15, and 20%. The *Amaranthus cruentus* (L) leaves, which were used in the present study, were grown under a controlled field trial in the North-West Province, South Africa. The mean temperatures around the area are above 22°C in summer and below 20°C in winter and lie at a latitude of 25.6200° S and a longitude of 27.9800° E. The aforementioned species were grown in September 2019, under dryland conditions, which receives a mean annual rainfall of less than 250 mm. Amaranth leaves were hand-harvested, thereafter, independently dried in a well-ventilated laboratory to obtain a constant weight, and milled through a 1 mm sieve into powder, by using a hammer mill, before for proximate analyses and being included in the formulated diets (Tables 1 and 2). Feed and water were provided *ad libitum* and the artificial heating and light of the poultry house were provided throughout the experiment.

**Table 1:** Proximate composition (g/100 g), gross energy (kcal/g) of *Amaranthus cruentus* leaf meal (ACLM).

Composition (g/100 g)	ACLM
DM	92.65
CP	23.23
CF	17.14
NDF	15.40
ADF	7.14
ADL	1.95
GE	14.50
EE	1.12
Starch	0.38
Ash	21.18

Values are means of duplicate analyzed amaranth leaves samples

**Table 2:** Ingredients and calculated analysis of experimental diets.

Amaranth leaf meal (ALM) inclusion levels (g/kg)					
Ingredients	0	5	10	15	20
Maize	40.00	40.00	40.00	40.00	40.00
Groundnut oil cake	20.00	18.00	15.00	12.00	10.00
Soya bean meal	8.00	8.00	8.00	8.00	8.00
Fish meal	2.00	2.00	2.00	2.00	2.00
Wheat offals	25.70	22.70	20.70	18.70	15.70
Bone meal	2.50	2.50	2.50	2.50	2.50
Limestone	0.50	0.50	0.50	0.50	0.50
Salt (NaCl)	0.50	0.50	0.50	0.50	0.50
Dl-Methionine	0.15	0.15	0.15	0.15	0.15
L-Lysine	0.15	0.15	0.15	0.15	0.15
Vit/Min Premix^†^	0.50	0.50	0.50	0.50	0.50
ALM	0.00	5.00	10.00	15.00	20.00
**Total**	100.00	100.00	100.00	100.00	100.00
**Calculated analysis**					
Crude protein (%)	20.00	20.00	20.00	20.00	20.00
Crude fibre (%)	4.52	4.57	4.71	4.70	4.73
Ether extract (%)	7.21	6.41	6.40	6.71	6.51
GE (kcal/100 g)	462.40	462.30	462.10	461.50	461.70
**Analyzed composition**					
Crude protein (%)	19.44	19.50	19.51	19.48	20.01
Crude fibre (%)	5.03	6.22	6.35	6.43	6.37
Ether extract (%)	7.61	7.65	7.80	7.81	7.79
GE (kcal/100g)	453.7	452.80	452.70	451.60	451.00

† The ingredients contained in the vitamin–mineral premix were as follows (per kg of diet): vitamin A 12000 IU, vitamin D3 3500 IU, vitamin E 30.0 mg, vitamin K 3 2.0 mg, thiamine 2 mg, riboflavin 6 mg, pyridoxine 5 mg, vitamin B12 0.02 mg, niacin 50 mg, pantothenate 12 mg, biotin 0.01 mg, folic acid 2 mg, Fe 60 mg, Zn 60 mg, Mn 80 mg, Cu 8 mg, Se 0.1 mg, Mo 1 mg, Co 0.3 mg, I 1 mg.

### 2.3 Data collection

#### 2.3.1 Growth performance

The initial live weight of each chicken was determined at the start of the experiment, thereafter, weekly weights were taken. The determined live weights were used to calculate the body weight gain of the chickens. The daily feed intake was determined by subtracting the weight of feed leftover from the total weight of the feed that was given to chickens daily and the difference was divided by the total number of chickens in each replicate. The feed conversion ratio was then calculated following the below-mentioned formulae: $Feed\;Conversion\;Ratio\;(FCR)=\frac{Feed\;intake\;(g)}{Bodyweight\;(g)}$

#### 2.3.2 Meat and bone characteristics

At the age of 42, three chickens per pen were slaughtered using the cervical dislocation method, following the recommendations of the University of Limpopo and the University of South Africa’s ethical guidelines. The birds were immersed in hot water to remove their feathers. Thereafter, carcass and meat parts’ weights were measured using an electronic weighing scale. Meat parts’ pH was measured using the digital pH meter (Crison, Basic 20 pH Meter) whereas, meat colour was measured using the HunterLab test (L*, a*, b*) system where L* is the lightness, a* is the redness, and b* is the yellowness. The shear force was done using Warner-Bratzler Shear Force procedures [13]. For sensory attributes, meat samples were frozen at -20°C and later allowed to be thawed for 24 h in a cooler room. A preheated oven was set to 160°C and was used to cook the breast meat. The meat sample of 1.5 cm thickness was boiled for approximately 50 min and turned over every 25 min. Tongs were used for turning to avoid piercing the meat, which could lead to moisture escaping. A taste panel of 25 assessors evaluated the meat for tenderness, juiciness, flavor, and overall acceptability by using a 5-point scale (Table 3).

**Table 3:** Evaluation score used by the sensory panel.

			Breast meat	
Score	Tenderness	Juiciness	Flavor	Overall acceptability
1	Too tough	Much too dry	Very bad flavor	Strongly dislike
2	Tough	Dry	Poor Flavour	Dislike
3	Neither tough nor tender	Neither dry nor juicy	Neither bad nor good flavor	Neither dislike nor like
4	Tender	Juicy	Good flavour	Like
5	Too tender	Too juicy	Very good flavor	Strongly like

For cooking loss, the chicken breast meats were weighed before and after roasting, and the percentage cooking loss was calculated as follows:

$$\%\;Cooking\;Loss=[(W_{0}‐W_{1})/W0]\;x\;100$$

Where W_0_ and W_1_ are the weights before and after cooking, respectively.

Furthermore, the tibia bone samples were cleaned from remnants and stored in isotonic saline at a temperature of -250°C. The geometric characteristics of the tibia bones were assessed by using the method of [14]. The content of minerals, such as Calcium and Potassium, was determined using the method of atomic absorption spectrometry [15].

#### 2.3.3 Statistical analysis

The statistical analysis was performed using the general linear model (GLM) procedure of [15]. All the data collected was analyzed using a completely randomized design. The mean was separated using the Duncan test at a significance level of p < 0.05. Furthermore, collected data was evaluated for linear and quadratic effects using polynomial contrasts.

The quadratic models were fitted to the experimental data by using the procedure of [16]. The response in optimum drumstick weight, breast pH, breast lightness and shear force, tibia and femur diameter of the broiler chickens, due to the inclusion of amaranth leaf meal, were modeled using the following quadratic equation:

$$Y=a+b_{1}\times +b_{2}\times_{2}$$

Where y = optimum, a = intercept; b = coefficients of the quadratic equation; × = *amaranthus* meal inclusion level and -b_1_/2b_1_ = × value for optimum response. The quadratic equation was the preferred model as it gives the optimum fit.

## 3. Results

### 3.1 Growth performance of broiler chickens fed varying inclusion levels of amaranth leaf meal

The results of the effect of amaranth leaf meal inclusion level on initial weight, final weight, average daily gain, average daily feed intake and feed conversion ratio of Ross 308 broiler chickens are presented in Table 4. Amaranth leaf meal inclusion level had no influence (p > 0.05) on final weight, average daily gain, average daily feed intake, and FCR of Ross 308 broiler chickens aged one to 42 days. Dietary inclusion of ALM had no linear (p > 0.05) or quadratic (p > 0.05) effects on initial weight, final weight, average daily gain, average daily feed intake, and feed conversion ratio of Ross 308 broiler chickens.

**Table 4:** Effect of amaranth inclusion leaf meal on initial weight (IW), final weight (FW) feed intake (ADFI), body weight gain (BWG) and feed conversion ratio (FCR) of Ross 308 broiler chickens.

	IW (g)	FW (g)	ADFI (g)	BWG(g)	FCR
ALM%					
0	45.75	1763.2	40.78	23.70	1.72
5	46.91	1834.9	43.69	22.76	1.92
10	44.41	1830.6	43.59	22.56	1.93
15	47.27	1794.3	41.98	23.44	1.79
20	44.91	1712.5	42.72	22.53	1.89
SEM	1.325	76.008	1.035	1.809	0.033
**P-value**					
Treatment	0.506	0.771	0.532	0.563	0.458
Linear	0.786	0.457	0.644	0.404	0.548
Quadratic	0.938	0.114	0.503	0.681	0.606

a, b Means in the same row not sharing a common superscript are different (p<0.05). Diets: ALM0% = a diet having no amaranth leaf meal inclusion, ALM5% = a diet having 5 g/kg of amaranth leaf meal inclusion, ALM10% = a diet having 10 g/kg of amaranth leaf meal inclusion. ALM15% = a diet having 15 g/kg of amaranth leaf meal inclusion. ALM20% = a diet having 20 g/kg of amaranth leaf meal inclusion. SEM: standard error of the mean.

### 3.2 Carcass and physiochemical characteristics

The findings of the influence of ALM inclusion levels on carcass and meat parts weights of Ross 308 broiler chickens are presented in Table 5. ALM inclusion levels had no effect (p > 0.05) on carcass, breast, or thigh weights of Ross 308 broiler chickens aged 42 days. However, ALM inclusion levels had an influence (p < 0.05) on the drumstick and abdominal fat weights of Ross 308 broiler chickens aged 42 days. Ross 308 broiler chickens on diet with a 20% ALM inclusion level, had heavier (p < 0.05) drumstick and abdominal fat weights than those offered 0, 5, 10, and 15% levels. Surprisingly, even though there was a significant difference between treatment on the drumstick and abdominal fat weights, no significant influence was observed at the linear (p > 0.05) or quadratic (p > 0.05) level.

**Table 5:** Effect of amaranth leaf meal inclusion on carcass meat part weights (g) of Ross 308 broiler chickens.

			Parameters		
ALM%	Carcass	Breast	Thigh	Drumstick	Abdominal Fats
0	1187.8	427.81	89.95	77.72^b^	4.16^b^
5	1344.8	463.83	94.27	81.30^ab^	7.39^ab^
10	1280.3	468.89	105.88	79.19^b^	6.33^ab^
15	1223.1	459.06	93.20	80.20^ab^	6.45^ab^
20	1202.4	453.48	90.81	98.95^a^	9.69^a^
**SEM**	75.785	22.967	8.128	4.280	1.221
**P-value**					
Treatment	0.655	0.807	0.653	0.041	0.012
Linear	0.715	0.437	0.980	0.147	0.104
Quadratic	0.432	0.121	0.384	0.181	0.354

^a, b, c,^ Means in the same row not sharing a common superscript are significantly different (p < 0.05). Diets: ALM 0% = a diet having no amaranth leaf meal inclusion, ALM 5% = a diet having 5 g/kg of amaranth leaf meal inclusion, ALM 10% = a diet having 10 g/kg of amaranth leaf meal inclusion. ALM 15% = a diet having 15 g/kg of amaranth leaf meal inclusion. ALM 20% = a diet having 20 g/kg of amaranth leaf meal inclusion. SEM: standard error of the mean.

Meat parts pH of Ross 308 broiler chickens which were fed ALM inclusion in their diets are presented in Table 6. ALM inclusion levels had no effect (p > 0.05) on the breast, thigh, and drumstick pH of Ross 308 broiler chickens aged 42 days. Dietary inclusion of ALM had no linear (p > 0.05) or quadratic (p > 0.05) effects on breast, thigh, and drumstick pH of Ross 308 broiler chickens.

**Table 6:** Effect of amaranth leaf meal inclusion on meat parts pH of Ross 308 broiler chickens.

		Parameters	
ALM%	Breast	Thigh	Drumstick
0	5.70	5.57	5.92
5	5.81	5.91	6.16
10	5.71	5.70	6.03
15	5.78	5.90	6.13
20	5.97	6.05	6.75
SEM	0.012	0.117	0.266
**P-value**			
Treatment	0.056	0.082	0.260
Linear	0.152	0.111	0.105
Quadratic	0.286	0.373	0.194

Diets: ALM 0% = a diet having no amaranth leaf meal inclusion, ALM 5% = a diet having 5 g/kg of amaranth leaf meal inclusion, ALM 10% = a diet having 10 g/kg of amaranth leaf meal inclusion. ALM 15% = a diet having 15 g/kg of amaranth leaf meal inclusion. ALM 20% = a diet having 20 g/kg of amaranth leaf meal inclusion. SEM: standard error of the mean.

The results of the effect of ALM inclusion levels on breast meat color of Ross 308 broiler chickens are presented in Table 7. ALM inclusion levels did not affect (p > 0.05) breast meat redness (a*) or yellowness (b*) of Ross 308 broiler chickens aged 42 days. ALM inclusion levels did influence (p < 0.05) the breast meat lightness (L*) of Ross 308 broiler chickens aged 42 days. Ross 308 broiler chickens which were fed with diets having 5 and 10% ALM inclusion levels, showed lighter (p < 0.05) breast meat color than those on diets with 0, 15, and 20%. Similarly, chickens on diets having 5, 10, and 15% ALM inclusion levels had similar (p < 0.05) breast meat color. Dietary inclusion of ALM had no linear (p > 0.05) or quadratic (p > 0.05) effects on breast meat color of Ross 308 broiler chickens.

**Table 7:** Effect of amaranth leaf meal inclusion on breast meat color of Ross 308 broiler chickens.

		Breast	
ALM%	L*	a *	b *
0	46.25^bc^	1.05	10.45
5	51.11^a^	0.04	8.87
10	50.16^a^	-0.99	9.12
15	49.14^ab^	-1.18	7.33
20	44.56^c^	-1.44	8.06
**SEM**	1.011	0.835	1.151
**P-value**			
Treatment	0.005	0.263	0.433
Linear	0.615	0.014	0.068
Quadratic	0.057	0.011	0.192

^a, b, c^ Means in the same row not sharing a common superscript are different (p < 0.05). Diets: ALM0% = a diet having no amaranth leaf meal inclusion, ALM 5% = a diet having 5 g/kg of amaranth leaf meal inclusion, ALM10% = a diet having 10 g/kg of amaranth leaf meal inclusion. ALM15% = a diet having 15 g/kg of amaranth leaf meal inclusion. ALM20% = a diet having 20 g/kg of amaranth leaf meal inclusion. SEM: standard error of the mean.

The effect of amaranth leaf meal inclusion levels on sensory attributes and shear force of Ross 308 broiler chickens are presented in Table 8. ALM inclusion levels had no effect (p > 0.05) on meat tenderness, juiciness, flavor, or overall acceptability of Ross 308 broiler chickens aged 42 days. ALM inclusion levels had an effect (p < 0.05) on breast meat cooking loss and shear force of Ross 308 broiler chickens aged 42 days. Ross 308 broiler chickens that were given diets with 0 and 10% ALM inclusion levels, had higher (p < 0.05) cooking loss than those on diets with 5, 15, and 20% of ALM. Ross 308 broiler chickens that were fed with diets having a 20% ALM inclusion level, had higher (p < 0.05) shear force values than those fed with diets containing 0, 5, 10, and 15% ALM inclusion levels. However, chickens fed with diets having 0 and 15% had similar (p > 0.05) shear force values. In addition, cooking loss and shear force of Ross 308 broiler chickens showed no linear or quadratic effects.

**Table 8:** Effect of amaranth leaf meal inclusion on sensory attributes, cooking loss, and shear force of Ross 308 broiler chickens.

		Parameters				
ALM%	Tenderness	Juiciness	Flavor	Overall acceptability	Cooking Loss	Shear force
0	3.000	3.00	3.33	3.33	26.88^a^	3.31^b^
5	3.000	3.00	3.00	3.33	22.58^d^	2.25^c^
10	3.000	3.33	3.33	3.33	23.86^c^	1.89^d^
15	3.000	3.00	3.33	3.00	23.95^b^	3.29^b^
20	3.000	3.33	3.00	3.00	23.86^c^	5.70^a^
**SEM**	0.000	0.211	0.258	0.258	0.012	0.012
**P-value**						
Treatment	0.521	0.580	0.737	0.737	<0.000	<0.000
Linear	0.182	0.308	0.638	0.058	0.528	0.266
Quadratic	0.143	0.667	0.857	0.190	0.848	0.066

Diets: ALM0% = a diet having no amaranth leaf meal inclusion, ALM5% = a diet having 5 g/kg of amaranth leaf meal inclusion, ALM10% = a diet having 10 g/kg of amaranth leaf meal inclusion. ALM15% = a diet having 15 g/kg of amaranth leaf meal inclusion. ALM20% = a diet having 20 g/kg of amaranth leaf meal inclusion. SEM: standard error of the mean.

### 3.3 Bone physical and chemical characteristics

The findings of the effect of ALM inclusion levels on bone characteristics of Ross 308 broiler chickens are presented in Table 9. At the age of 21 days, ALM inclusion levels did not influence (p > 0.05) tibia and femur lengths, weights, or seedor indexes of Ross 308 broiler chickens. Ross 308 broiler chickens that were fed with diets having a 15% ALM inclusion level, had a wider (p > 0.05) tibia diameter than chickens on diets having 0, 5, 10, and 20% ALM inclusion levels. However, chickens on diets with 5 and 15% ALM inclusion levels, had similar (p > 0.05) tibia diameters. Ross 308 broiler chickens that were fed with diets having 5 and 15% ALM inclusion levels, had greater (p < 0.05) femur diameters than those on diets having 0, 10, and 20% ALM inclusion levels. Similarly, chickens on diets having 5 and 15% ALM inclusion levels, had the same (p < 0.05) femur diameters. At 42 days of age, ALM inclusion levels had no effect (p > 0.05) on tibia weight, diameter, or seedor index, nor on femur length, weight, diameter, or seedor index of Ross 308 broiler chickens aged 42 days. However, Ross 308 broiler chickens fed with diets with a 15% ALM inclusion level, showed longer (p < 0.05) tibia lengths than those given diets having 0, 5, 10, and 20% ALM inclusion levels. However, chickens on diets having 0, 5, 10, and 15% levels had similar (p < 0.05) tibia lengths. At the age of 21 days, tibia weight showed linear (p = 0.002) and quadratic (p = 0.013) increases with an increase in ALM in the diets. However, femur weight had a linear (p = 0.040) effect with an increase in ALM in the diets. Whereas the femur seedor index, had a linear (p = 0.002) and quadratic (p = 0.002) increases with an increase in ALM in the diets.

**Table 9:** Effect of amaranth inclusion leaf meal on bone characteristics of Ross 308 broiler chickens.

	Tibia				Femur			
	Length	Weight	Diameter	S index	Length	Weight	Diameter	S index
**ALM**								
**21 days**								
**0**	5.25	2.99	3.82^c^	0.57	3.13	1.16	4.96^b^	0.37
**5**	5.00	2.77	5.05^ab^	0.49	3.30	1.20	5.93^a^	0.35
**10**	5.13	2.50	4.00^c^	0.54	3.50	1.15	4.94^b^	0.33
**15**	4.92	2.25	5.28^a^	0.46	3.25	0.93	6.17^a^	0.29
**20**	5.00	2.19	4.85^b^	0.43	3.00	0.85	5.67^ab^	0.28
**SEM**	0.192	0.314	0.129	0.049	0.173	0.165	0.238	0.046
**P-value**								
Treatment	0.773	0.356	<0.000	0.855	0.357	0.448	0.006	0.761
Linear	0.184	0.002	0.330	0.063	0.671	0.040	0.427	0.002
Quadratic	0.396	0.013	0.649	0.260	0.088	0.076	0.752	0.002
**42 days**								
**0**	9.31^ab^	10.30	7.00	0.91	6.91	7.72	8.25	0.90
**5**	8.88^ab^	10.16	7.25	0.91	6.51	7.09	7.75	0.95
**10**	9.10^ab^	9.38	6.49	0.97	6.88	7.22	7.75	0.96
**15**	9.53^a^	9.54	6.38	1.00	6.91	7.05	6.75	0.98
**20**	8.84^b^	8.99	7.08	0.99	6.97	6.89	7.31	1.02
**SEM**	0.206	0.627	0.373	0.059	0.120	0.473	0.375	0.063
**P-value**								
Treatment	0.144	0.566	0.408	0.763	0.103	0.776	0.112	0.821
Linear	0.273	0.020	0.632	0.031	0.454	0.069	0.097	0.005
Quadratic	0.369	0.125	0.621	0.145	0.628	0.207	0.275	0.050

a, b, c Means in the same row not sharing a common superscript are different (p<0.05). Diets: ALM0% = a diet having no ALM inclusion, ALM5% = a diet having 5 g/kg of ALM inclusion, ALM10% = a diet having 10 g/kg of ALM inclusion. ALM15% = a diet having 15 g/kg of ALM inclusion. ALM20% = a diet having 20 g/kg of ALM inclusion. SEM: standard error of the mean.

The bone minerals of Ross 308 broiler chickens, aged 21 and 42 days are presented in Table 10. ALM inclusion levels had an effect (p < 0.05) on tibia and femur Ca and P in Ross 308 broiler chickens aged 21 and 42 days, respectively. At the age of 21 days, Ross 308 broiler chickens which were fed with diets having a 5% ALM inclusion level, had higher (p < 0.05) tibia calcium (Ca), phosphorus and femur Ca than those fed with diets having 0, 10, 15, and 20% ALM inclusion levels. Ross 308 broiler chickens that were fed with diets having a 15% ALM inclusion level, had higher (p < 0.05) femur P than those fed with diets containing 0, 10, and 20% ALM inclusion levels. At 42 days of age, Ross 308 broiler chickens which were fed with diets having a 5% ALM inclusion level, had higher (p < 0.05) tibia Ca and femur P than those fed with diets containing 0, 10, 15, and 20% ALM inclusion levels. Ross 308 broiler chickens that were fed with control diets (ALM 0%) had higher (p < 0.05) tibia P than those fed with diets having 5, 10, 15, and 20% ALM inclusion levels. Ross 308 broiler chickens that were fed with diets having a 15% ALM inclusion level, had higher (p < 0.05) femur Ca than those fed with diets having 0, 5, 10, and 20% ALM inclusion levels. Moreover, at the age of 21- and 42-days, bone minerals of Ross 308 broiler chickens showed no significant linear or quadratic effect.

**Table 10:** Effect of amaranth inclusion leaf meal on bone minerals of Ross 308 broiler chickens.

Tibia			Femur	
	Ca	P	Ca	P
**ALM**				
**21 days**				
**0**	25.30^e^	9.98^d^	22.15^e^	11.05^e^
**5**	31.32^a^	12.26^a^	24.64^a^	11.90^b^
**10**	30.51^c^	11.62^b^	22.15^d^	11.49^c^
**15**	30.77^b^	9.00^e^	22.30^c^	12.46^a^
**20**	25.91^d^	10.86^c^	23.77^b^	11.35^d^
**SEM**	0.012	0.011	0.012	0.011
**P-value**				
Treatment	<0.000	<0.000	<0.000	<0.000
Linear	0.954	0.768	0.842	0.581
Quadratic	0.093	0.882	0.980	0.501
**42 days**				
**0**	21.49^e^	12.42^a^	29.87^e^	10.23^b^
**5**	24.98^a^	11.36^b^	32.63^c^	11.36^a^
**10**	23.39^c^	11.34^b^	31.21^d^	9.01^c^
**15**	24.65^b^	9.85^d^	34.38^a^	10.23^b^
**20**	22.81^d^	11.34^b^	33.44^b^	9.01^d^
**SEM**	0.012	0.012	0.011	0.012
**P-value**				
Treatment	<0.000	<0.000	<0.000	<0.000
Linear	0.676	0.251	0.291	0.315
Quadratic	0.391	0.318	0.201	0.651

a, b, c, d, e Means in the same row not sharing a common superscript are different (p < 0.05). Diets: ALM0% = a diet having no ALM inclusion, ALM5% = a diet having 5 g/kg of ALM inclusion, ALM10% = a diet having 10 g/kg of ALM inclusion. ALM15% = a diet having 15 g/kg of ALM inclusion. ALM20% = a diet having 20 g/kg of ALM inclusion. SEM: standard error of the mean.

## 4. Discussion

The results of the current study showed that amaranth leaf meal (ALM) inclusion did not affect initial weight, final weight, average daily body weight gain, average daily feed intake, and feed conversion ratio of Ross 308 broiler chickens aged one to 42 days. The findings of the current study are in agreement with the results of [9] who did not observe any effect on growth performance when broiler chickens were fed diets having up to 8% amaranth feed mixture. On contrary, [8, 10] reported that the addition of 8% amaranth to the diet mixture reduced the bodyweight of the chickens. Moreover, [17] reported that amaranth leaf meal inclusion in broiler chicken diets has significant differences though their FCR values were higher than the values of the current study. The reported difference, with the current study might be due to the fact that the amaranth used in this study, was perhaps harvested before the accumulation of secondary metabolites and also may be due to the different climatic conditions in which it was planted. ALM inclusion levels did not affect carcass, breast, or thigh weight of Ross 308 broiler chickens aged 42 days. Results of the present study are in agreement with the results of [8, 9], who reported that no difference was observed when broiler chickens were given an amaranth based diet. In the contrary, [10] observed an increase in the breast muscle percentage in carcasses of chickens fed with 8% amaranth feed mixture. However, the authors stated that an increase in breast muscle might be due to 3% flax oils, which was part of their formulated diet, and which was not present in the current study. Ross 308 broiler chickens on diets having 5, 15, and 20% amaranth leaf meal inclusion levels, had heavier drumstick weights than those on diets having 0, 5, 10, and 15% levels. In the contrary, [8] reported no effect on abdominal fat yield when using an 8% amaranth inclusion level. [11] reported an increase in abdominal fats when chickens were fed diets having an amaranth leaf meal inclusion of up to 10%. The reported difference, with the current study might be due to the fact that the amaranth used in this study, was perhaps harvested before the accumulation of secondary metabolites and also may be due to the different climatic conditions in which it was raised.

ALM inclusion levels had no effect on the thigh and drumstick pH of Ross 308 broiler chickens aged 42 days. ALM inclusion levels influenced the breast meat pH of Ross 308 broiler chickens aged 42 days. Ross 308 broiler chickens which were fed with diets having a 20% ALM inclusion level, had higher breast meat pH values than those on diets having 0, 5, 10, and 15% levels. According to [18], the normal pH of breast meat ranges between 5.7 and 5.96. The pH values obtained in the present study, are within the normal values as stated by [18]. ALM inclusion levels had no effect on breast meat redness (a*) or yellowness (b*) of Ross 308 broiler chickens aged 42 days. ALM inclusion levels affected breast meat lightness (L*) of Ross 308 broiler chickens aged 42 days. Ross 308 broiler chickens on diets with 5 and 10% ALM inclusion levels, had a lighter breast meat colour than those fed diets having 0, 15, and 20% levels. According to [19], the light colour is an indication of the meat’s freshness and directly influences the consumer’s final purchase decision. In the present study, the reason why low levels of ALM inclusion resulted in lighter breast meat colour, than when ALM inclusion was increasing, might be due to the high chlorophyll present in green leaves and, as ALM was increasing, the carotene levels were increasing. Amaranth leaf meal inclusion levels had no effect on meat tenderness, juiciness, flavour, or overall acceptability of Ross 308 broiler chickens aged 42 days. However, the results of the current study are in contrast with those of [10]. The authors reported that amaranth supplementation in chicken diets resulted in a significant difference in sensory analysis due to the improved aroma of the meat. However, [20] reported an improvement in the juiciness of the chicken meat when the chickens were given a diet supplemented with a 4% amaranth as an additive.

In the current study, ALM inclusion levels had an effect on breast meat cooking loss and shear force of Ross 308 broiler chickens aged 42 days. Ross 308 broiler chickens fed with diets having 0 and 10% ALM inclusion levels, had higher cooking loss than those on diets having 5, 15, and 20% of ALM inclusion levels. However, according to [10], there was a decrease in the cooking loss in the breast meat of chickens fed with about 8% amaranth. Nevertheless, [20], reported that no negative influence was observed on the physicochemical properties of the breast meat of chickens fed with amaranth. In the present study, chickens on diets having a 5% ALM level, had higher shear force values than those on diets with a 10% ALM inclusion level. However, the values obtained in the current study have been considered to be acceptable by consumers [21–23]. Yet, according to [24], shear force values of cooked breast meat change depending on the age of the broilers. Moreover, the authors stated that between 3.46 to 6.41 shear force values of breast meat, can be considered moderately tender. Though, shear force values, in the current study, ranged from 1.89 to 5.70 which means that the meat can be considered to be tender to consumers.

In improved chickens, like Ross 308 broilers, skeletal abnormalities and fractures of bone are the results of a higher muscle to the bone ratio [25]. Moreover, bone conditions, such as strength, weight, and length, are directly related to the bioavailability of minerals, such as calcium (Ca) and phosphorus (P) at the tissue level [26].

In the current study, ALM inclusion levels did not affect tibia length, weight, or seedor index, nor on femur length, weight, or seedor index of Ross 308 broiler chickens aged 21 days. However, ALM inclusion levels affected tibia diameters of Ross 308 broiler chickens aged 21 days. Ross 308 broiler chickens, on diets having a 15% ALM level, had greater tibia diameters than those on diets having 0, 5, 10, and 20% ALM inclusion levels. ALM inclusion levels affected femur diameters of Ross 308 broiler chickens at 21 days. Ross 308 broiler chickens on diets with 5 and 15% ALM inclusion levels, had greater femur diameters than those on diets with 0, 10, and 20% ALM inclusion levels. To the best of our knowledge, there are no studies available in the literature on the use of amaranth leaf meal on bone characteristics in Ross 308 broiler chickens. For this reason, all the comparisons with the available data in literature had to do with other leaf meals, from other plant species, which possess almost the same nutritional content as vegetable amaranth. The findings of the current study disagree with the results of [6] who reported that *Moringa oleifera* leaf meal did not affect tibia bone characteristics. However, according to [7], chickens fed with diets containing *Moringa oleifera*, showed an increase in bone weight and ash percentage. High ash percentage is a good indicator of high mineral content in bones, which might be due to bioactive compounds found in leaves [27, 28]. [29] propose that another possible reason for improved bone parameters might be the presence of flavonoids found in *Moringa oleifera*. Flavonoids have the ability to inhibit the activities of osteoclasts and to promote bone mineralization through protein synthesis in the bones and osteoblast synthesis [29].

## 5. Conclusion

In conclusion, amaranth leaf meal can be included in broiler chickens without having any adverse effect on the chickens’ performance. Nutrient utilization and affected parameters showed in favour of an inclusion level of 5, 10, and 15% levels in broiler diets.
